# Artificial intelligence-enhanced assessment of fundamental motor skills: validity and reliability of the FUS test for jumping rope performance

**DOI:** 10.3389/frai.2025.1611534

**Published:** 2025-08-04

**Authors:** Hubert Makaruk, Jared M. Porter, E. Kipling Webster, Beata Makaruk, Paweł Tomaszewski, Marta Nogal, Daniel Gawłowski, Łukasz Sobański, Bartosz Molik, Jerzy Sadowski

**Affiliations:** ^1^Faculty of Physical Education and Health in Biala Podlaska, Józef Piłsudski University of Physical Education in Warsaw, Warsaw, Poland; ^2^Department of Kinesiology, Recreation, and Sport Studies, University of Tennessee, Knoxville, TN, United States; ^3^Faculty of Physical Education, Józef Piłsudski University of Physical Education in Warsaw, Warsaw, Poland; ^4^Artificial Intelligence Department, DG Consulting, Wrocław, Poland; ^5^Department of Research in Artificial Intelligence, Instat sp. z o.o., Wrocław, Poland; ^6^Faculty of Rehabilitation, Józef Piłsudski University of Physical Education in Warsaw, Warsaw, Poland

**Keywords:** motor competence, fundamental movement skills, machine learning, mobile application, physical education

## Abstract

**Introduction:**

Widespread concerns about children’s low fundamental motor skill (FMS) proficiency highlight the need for accurate assessment tools to support structured instruction. This study examined the validity and reliability of an AI-enhanced methodology for assessing jumping rope performance within the Fundamental Motor Skills in Sport (FUS) test.

**Methods:**

A total of 236 participants (126 primary school students aged 7–14; 110 university sports students aged 20–21) completed jumping rope tasks recorded via the FUS mobile app integrated with an AI model evaluating five process-oriented performance criteria. Concurrent validity and inter-rater reliability were examined by comparing AIgenerated assessments with scores from two expert evaluators. Intra-rater reliability was also assessed through reassessment of video trials after a 3-week interval.

**Results:**

Results revealed excellent concurrent validity and inter-rater reliability for the AI model compared with expert ratings (ICC = 0.96; weighted kappa = 0.87). Agreement on individual criteria was similarly high (Cohen’s kappa = 0.83–0.87). Expertadjusted AI scores further improved reliability (ICC = 0.98). Intrarater reliability was also excellent, with perfect agreement for AIgenerated scores (ICC = 1.00; kappa = 1.00).

**Conclusions:**

These findings demonstrate that AI-based assessment offers objective, reliable, and scalable evaluation, enhancing accuracy and efficiency of FMS assessment in education and research.

## Introduction

Assessing and improving movement proficiency during childhood is crucial for supporting optimal physical development and building the foundational skills necessary for lifelong physical activity (Logan et al., 2015; Stodden et al., 2008). Fundamental motor skills (FMS), including jumping, running, and throwing, form the foundation for more complex motor tasks, facilitating children’s effective participation in diverse sports and physical activities (Logan et al., 2018). A large body of research indicates that proficiency in FMS is associated with enhanced physical fitness and a reduced risk of lifestyle-related health issues, including obesity and cardiovascular conditions (Barnett et al., 2022; Robinson et al., 2015; Stodden et al., 2008). Conversely, children with insufficient FMS often face barriers for participation in sports and social play, which can negatively affect their physical, emotional, and social development (Robinson et al., 2015).

Process-oriented assessments are widely recognized as the paramount benchmark for evaluating FMS, offering detailed insights into how movements are performed (Logan et al., 2017; Watanabe et al., 2024). Unlike product-oriented tools that emphasize measurable outcomes such as distance or speed, process-oriented evaluations focus on coordination, control, and other qualitative factors, providing deeper insights into the mechanics and efficiency of motor performance (Logan et al., 2018). These tools are particularly valuable for identifying deficits in specific movement components, which are essential for advanced skill proficiency. For instance, O’Brien et al. (2016) highlighted that poor execution of fundamental behavioral components, such as bending the knees and extending the arms during take-off, was a common reason for failure in both vertical and horizontal jumps.

Despite their predictive value for assessing FMS proficiency, implementing process-oriented assessments poses substantial challenges, even for trained experts and researchers (Hulteen et al., 2018; Hulteen et al., 2023; Ward et al., 2020). Ensuring validity and reliability requires extensive training and strict adherence to protocols, making the process resource-intensive and time-consuming (Lander et al., 2015). Variability in scoring frequently arises from subjective interpretations of movement quality, particularly during real-time assessments, where observers must simultaneously track multiple criteria, compromising accuracy (Barnett et al., 2014; Ward et al., 2020). The issue becomes more pronounced with less experienced evaluators, emphasizing the need to improve training procedures, assessment frameworks, and clearly defined evaluation criteria (Lander et al., 2015; Palmer and Brian, 2016). Additionally, inherent constraints in process-oriented assessments limit the number of performance criteria that human observers can reliably evaluate. For example, widely-used tools, such as the Test of Gross Motor Development (Webster and Ulrich, 2017), address this limitation by removing criteria during an item-analysis phase if they cannot be consistently scored by human observers. However, this practice may result in essential movement characteristics remaining unassessed. Barnett et al. (2014) illustrate this problem, noting that inadequately defined criteria, such as not specifying elbow flexion, can cause incorrect movements (e.g., a “fling” or “sling”) to be incorrectly scored as correct. Similarly, tools like the Victorian Fundamental Motor Skill Manual (Walkley et al., 1996) provide partial guidance but fail to fully represent the complexity and subtlety inherent in FMS, further complicating accurate assessment. These limitations collectively underscore the need for more comprehensive assessment strategies.

In many countries, early education (classroom) and physical education (PE) teachers are responsible for monitoring FMS development as part of the curriculum (Lander et al., 2016; Makaruk et al., 2024b). However, many teachers lack confidence in utilizing process-oriented tools, which often demand a deeper understanding of human movement and FMS development (Bourke et al., 2024; Harris et al., 2011; Lander et al., 2015). This knowledge gap can lead to inconsistent evaluations and an over-reliance on simplified tools that fail to capture the complexities of motor skills (Morley et al., 2019). Additionally, limited training and professional development opportunities contribute to these obstacles, leaving many teachers unprepared to effectively assess and address movement skill deficiencies within the constraints of their teaching schedules (Draper et al., 2019). Research highlights that teachers often prioritize instructional activities over assessments due to the significant time and logistical burdens associated with administering traditional FMS tools, particularly in large class settings (Draper et al., 2019; Morley et al., 2019). Morley et al. (2019) noted that these tools are not only time-intensive but also require specialized expertise, making their integration into daily teaching routines difficult. Furthermore, limited access to updated FMS testing methodologies and appropriate resources frequently forces teachers to rely on simplified or outdated tools, which fail to capture the full scope of movement skills (Bourke et al., 2024; Foweather et al., 2018; Morley et al., 2019). As a result, assessments are often infrequent and incomplete, reducing the effectiveness of identifying and addressing movement deficiencies in students.

Addressing these challenges requires innovative solutions that preserve the depth and accuracy of process-oriented assessments (Bisi et al., 2017; Hulteen et al., 2020; Ward et al., 2017) while enhancing their feasibility. Digital technology, such as tablet-based applications, has shown great potential in this regard and is well-received by primary school teachers. These tools provide functionalities like video recording and analysis, enabling educators to capture and review children’s performances effectively (Browne, 2015; Draper et al., 2019). Digital tools streamline the assessment process and provide visual evidence of movement execution, enabling more accurate identification of skill deficiencies. Embedded video demonstrations further support teachers with limited expertise, offering references for skill performance and guidance for conducting assessments (Foweather et al., 2018; Makaruk et al., 2024a; O’Loughlin et al., 2013). One such example is Meu Educativo®, a platform designed to evaluate FMS using an expert-validated checklist and rating system (Garbeloto et al., 2024). The tool prioritizes ease of use, maintaining reliability with inter-rater reliability ranging from 0.63 to 0.93 and intra-rater reliability from 0.46 to 0.94. Another example is the FUS test app, which optimizes the evaluation process by providing teachers and researchers with tools to efficiently record, analyze, and score performances (Makaruk et al., 2024a). Validation studies demonstrated strong concurrent validity (*r* = 0.92–0.96) and excellent intra-rater reliability (ICC > 0.91), supporting its use as a reliable tool for assessing FMS.

Emerging technologies like artificial intelligence (AI) build on advancements in digital tools, may offer transformative potential to enhance the accessibility, reliability, and accuracy of FMS assessments (Vandevoorde et al., 2022). AI-powered tools can automate labor-intensive tasks, such as video analysis and scoring, reducing the need for extensive training and systematic observation scoring. For example, Zhang et al. (2024) validated the precision of AI-powered tools in analyzing biomechanical markers, effectively detecting subtle deficits in balance and coordination that are often overlooked with conventional feedback from supervisors. Similarly, Sganga et al. (2023) confirmed the effectiveness of integrating smartphone-based inertial motion units with AI algorithms to accurately estimate the relative displacement between the center of mass and the center of pressure. Another study (Hajihosseini et al., 2022) established the feasibility of automated assessments for FMS like overhand throwing. The AI-powered system, which employs wearable inertial measurement units and the ‘k-nearest neighbor’ algorithm, reduced scoring time from 5 min to less than 30 s while maintaining high accuracy, with classification rates ranging from 76 to 93% across four specific criteria. AI-based tools have proven effective in evaluating key rope-jumping performance parameters, including jumping pace, flight time, touchdown time, and jump height, achieving excellent reliability (ICC > 0.9) (Yu and Hu, 2022). Collectively, these advancements underscore the transformative potential of AI in bridging the gap between traditional, resource-intensive methods and scalable solutions for fostering motor skill learning and development.

Despite increasing use of digital tools in PE, there remains a pressing need to validate AI-powered systems capable of delivering standardized, efficient, and scalable FMS assessments. The primary aim of this study was to evaluate the validity and reliability of an AI-enhanced methodology for assessing jumping rope performance within the FUS test framework. This approach was intended to address key limitations of traditional process-oriented methods, such as scoring variability, time constraints, and the need for specialized expertise, by examining the AI model’s capacity to deliver objective and consistent movement analysis. We hypothesized that integrating AI systems could standardize FMS evaluations, reduce logistical barriers, and support broader implementation in both educational and research settings. These assumptions underpin a broader vision of improving the accessibility and quality of motor skill assessments while advancing evidence-based practice through data-driven innovation.

## Methods

### Participants

A total of 236 students participated in this study, comprising 126 primary school students aged 7–14 years (54% female) and 110 university sports students aged 20–21 years (45% female). Older participants were included to validate the assessment protocol across a broader age and skill spectrum, extending beyond the originally targeted age range of the FUS test. Participants were eligible if they were actively enrolled in physical activity-related educational programs offered by their institution and had no medical conditions, musculoskeletal injuries, or neurological disorders affecting jumping performance. Written informed consent was obtained from all participants or their legal guardians, and the study protocol was approved by the institutional Research Ethics Committee.

### Apparatus and technology

The FUS test app, developed for use on mobile phones and tablets, was employed to assess proficiency in six sports-related tasks, including jumping rope performance. The app facilitated video recording, analysis, and scoring based on predefined performance criteria. To evaluate the task of jumping rope, an AI-driven assessment system was incorporated to provide an automated and objective evaluation of movement proficiency. The AI assessment utilized the MoveNet model, an open-source deep neural network developed by Google (TensorFlow, 2021). The model tracked the horizontal and vertical coordinates of 17 body parts at a frequency of 10 frames per second, providing precise motion data for detailed analysis. The anatomical landmarks included the nose, left and right eyes, left and right ears, left and right shoulders, left and right elbows, left and right wrists, left and right hips, left and right knees, and left and right ankles.

Jump rope proficiency was evaluated by analyzing five key movement characteristics aligned to the FUS (reference) scoring criterion, each assessed using a machine learning model specifically developed for the corresponding criterion: continuity (criterion 1), rhythmicity (criterion 2), arm and wrist positioning (criterion 3), hip and knee flexion (criterion 4), and central positioning (criterion 5). Continuity was modeled using the Extra Trees Classifier (Geurts et al., 2006), an ensemble learning algorithm that analyzed temporal patterns to identify whether jumps were performed continuously without interruptions. Rhythmicity, representing the consistency and timing of jumps, was evaluated with a Gradient Boosting Classifier (Friedman, 2001) designed to detect deviations in timing across consecutive cycles. Arm and wrist positioning was assessed using another Gradient Boosting Classifier, which analyzed the spatial and temporal precision of arm and wrist movements during the swinging phase. Hip and knee flexion was modeled using a Multilayer Perceptron Neural Network Classifier (Rumelhart et al., 1986), a network capable of detecting subtle variations in joint angles. Finally, central positioning was assessed with a Gradient Boosting Classifier, which evaluated spatial alignment and postural control to ensure jumps were executed within the designated area while maintaining an upright trunk position. Each of these models was designed to provide automated, objective assessments of movement proficiency, scoring individual criteria on a binary scale where 1 indicated correct execution and 0 indicated incorrect execution. In cases where the AI system failed to detect the participant’s movements with sufficient confidence, such as poor video quality issues or obstruction, a score vector of (−1, −1, −1, −1, −1) was returned, indicating a failed recognition attempt. The AI model assessment utilized open-source libraries, including TensorFlow (2021), licensed under Apache 2.0, and scikit-learn (Pedregosa et al., 2011), licensed under the BSD License.

Data collection was conducted using a Lenovo Tab P11 (2nd Gen) tablet, equipped with a 13 MP high-resolution camera capable of recording 1080p HD videos at 30 frames per second. The tablet’s 2,000 × 1,200 resolution screen provided high-quality playback, supporting accurate video analysis.

### Procedure

The jumping rope task in the FUS test required participants to perform rhythmic and continuous jumps over the rope for 10 s. The app, however, allowed for a 15-s video recording, capturing both the preparation phase (1–2 s) and the task execution. The following standardized procedure was implemented to ensure consistency across trials. At the start of each trial, the participant stood directly in front of the test administrator, positioned approximately 4 m from the camera, which was aligned to face the center of the “X” marked on the floor. The administrator provided clear instructions: “Jump to the rhythm of the rope hitting the ground,” and ensured the participant was prepared to proceed. Upon confirmation, the administrator activated the recording function in the application and instructed the participant to begin the task by saying “Go.” The app automatically stopped recording after 15 s, ensuring standardized durations across all trials.

Before beginning the warm-up trial, participants were instructed to adopt an upright stance, holding the handles of the rope behind their body. Arms were positioned close to the trunk, with elbows bent and externally abducted. The length of the rope was adjusted according to the participant’s height, ensuring that, when folded in half, it extended from the floor to the shoulder. All jumps were performed on a flat wooden surface to ensure safety and consistency in testing conditions. To preserve optimal video analysis and minimize potential distractions, no other individuals were permitted near or in the background of the participant during the task. Additionally, the background was required to provide sufficient contrast with the participant’s clothing (e.g., light-colored clothing against a dark background) to enhance visibility and ensure accuracy in movement assessment.

To ensure clarity and correct execution, the test supervisor presented the task before the trials commenced. Participants observed the demonstration from a position directly in front of the supervisor. Following the instructional phase, each participant completed a warm-up trial to familiarize themselves with the task. Subsequently, two test trials were conducted, with a minimum rest interval of 3 min between trials to mitigate fatigue. No feedback was provided during testing.

The criteria for jumping rope are as follows: criterion 1. jumps are performed continuously (without stopping); criterion 2. jumps are rhythmic and single, with short ground contact time and landing on the ball of the feet; criterion 3. arms are bent and held close to the trunk, and the rope is moved using the rotation of forearms and wrists; criterion 4. knees and hips are slightly bent during flight and landing; criterion 5. jumps are performed vertically with jumps initiating in the same designated area, with the trunk upright, feet parallel at a hip width apart.

Each criterion was scored as “1” if met or “0” if unmet, with points awarded only when performance clearly satisfied the respective criterion. The higher-scoring attempt was considered for further analysis.

### Concurrent validity and inter-rater reliability between FMS experts and AI model

In this study, concurrent validity evaluated how well the AI model replicated expert evaluations, recognized as the benchmark for jumping rope proficiency. Inter-rater reliability analyzed the consistency of scores between the AI model and human assessors. Both psychometric evaluations employed the same statistical measures. Two experienced assessors independently scored 236 video-recorded performances, resolving disagreements through a consensus process to ensure accuracy. Prior to consensus, the assessors achieved at least 90% agreement on total scores. A third assessor utilized the AI model to independently evaluate the same performance criteria. The AI-generated scores, produced automatically by the software’s predefined algorithms, were then directly compared with scores assigned by the human experts. This comparison allowed for an evaluation of both the validity and reliability of the AI assessment system.

### Inter-rater reliability between FMS experts correcting AI-generated scores

A second analysis was conducted to assess the inter-rater reliability of the AI model after adjustments by human experts. Two independent evaluators reviewed the AI-generated scores for the same set of video-recorded performances (*n* = 236). When the AI-generated scores differed from the expert’s judgment, the experts manually adjusted (corrected) these scores to align them with their expert evaluation. Corrections were made only when the expert was clearly satisfied that the AI-generated point did not accurately reflect the actual observed performance.

### Intra-rater reliability of the AI model and intra-rater reliability between FMS experts correcting AI-generated scores

The intra-rater reliability of the AI model, operated independently by an expert, and the AI model corrected by two expert raters was independently evaluated by comparing scores from the initial and follow-up assessments of the same video-recorded jumping rope performances (*n* = 236) after a three-week interval. Corrections were applied only when the expert was fully confident that a point should or should not be awarded for a specific criterion.

### Statistical analysis

Descriptive statistics were reported as means and standard deviations for all variables. Concurrent validity, inter-rater and intra-rater reliability were assessed using the percentage of observed agreements, Cohen’s kappa coefficients for individual criteria, and weighted kappa coefficients for total points, along with intraclass correlation coefficients (ICCs). Cohen’s kappa values were interpreted based on the classification proposed by Landis and Koch (1977), where values <0 indicate poor agreement, 0.01–0.20 indicate slight agreement, 0.21–0.40 indicate fair agreement, 0.41–0.60 indicate moderate agreement, 0.61–0.80 indicate substantial agreement, and 0.81–1.00 indicate almost perfect agreement. ICC values were interpreted as follows: values <0.5 indicate poor reliability, 0.5–0.75 indicate moderate reliability, 0.75–0.9 indicate good reliability, and >0.9 indicate excellent reliability (Koo and Li, 2016). For both Cohen’s kappa and ICC values, 95% confidence intervals (CIs) were calculated to provide a measure of precision. The statistical significance threshold was set at alpha = 0.05 for all analyses. Data were analyzed using SPSS Statistics version 27 for Windows (SPSS Inc., Chicago, USA).

## Results

### Concurrent validity and inter-rater reliability

The total scores for the jumping rope assessment were nearly identical between the FMS experts (3.12 ± 1.80) and the AI model (3.12 ± 1.77). As presented in Table 1, the observed agreement for individual performance criteria ranged from 92.8% (criteria 4 and 5) to 94.5% (criterion 2). Cohen’s kappa values ranged from 0.83 (criterion 5) to 0.87 (criterion 2), indicating almost perfect inter-rater agreement. The total score demonstrated strong consistency between raters, with an observed agreement of 77.1%, a Cohen’s kappa of 0.87 (95% CI: 0.84–0.91), and an excellent ICC of 0.96. Minor discrepancies between expert and AI scores occurred evenly across all criteria, with no consistent direction of bias.

**Table 1:** Concurrent validity and inter-rater reliability for jumping rope assessment between FMS experts and AI model (*n* = 236).

Jumping rope	Percentage of observed agreements (%)	Cohen’s kappa (95% CI)
Criterion 1	93.2	0.86 (0.79–0.92)
Criterion 2	94.5	0.87 (0.81–0.94)
Criterion 3	93.6	0.87 (0.80–0.93)
Criterion 4	92.8	0.86 (0.79–0.92)
Criterion 5	92.8	0.83 (0.76–0.91)
*Total score*	77.1	0.87 (0.84–0.91)

### Concurrent validity and inter-rater reliability between FMS experts correcting AI-generated scores

Table 2 summarizes the results for AI-generated scores adjusted by FMS experts. Observed agreements were consistently high, ranging from 96.6% (criterion 4) to 98.7% (criterion 1). Cohen’s kappa coefficients varied between 0.93 (criteria 4 and 5) and 0.97 (criterion 1), consistently reflecting almost perfect agreement. For the total score, observed agreement was 89.0%, Cohen’s kappa was 0.94, and the ICC for the total score was excellent at 0.98 (95% CI: 0.98–0.99). The two evaluators showed strong consistency across all criteria, with minor discrepancies evenly distributed and no consistent scoring bias identified. Differences between evaluators ranged from 3 to 8 cases per criterion, with total score agreement in 210 of 236 assessments.

**Table 2:** Inter-rater reliability for jumping rope assessment between experts correcting AI-generated scores (*n* = 236).

Jumping rope	Percentage of observed agreements (%)	Cohen’s kappa (95% CI)
Criterion 1	98.7	0.97 (0.94–1.00)
Criterion 2	97.5	0.94 (0.90–0.99)
Criterion 3	97.5	0.94 (0.90–0.99)
Criterion 4	96.6	0.93 (0.88–0.98)
Criterion 5	97.0	0.93 (0.88–0.98)
*Total score*	89.0	0.94 (0.92–0.97)

### Intra-rater reliability for AI model and intra-rater reliability between FMS experts correcting AI-generated scores

The AI model exhibited perfect consistency, achieving 100% observed agreement and a kappa coefficient of 1.00 across all criteria and the total score (Table 3). After correction by FMS experts, intra-rater agreement remained high, with observed agreements ranging from 98.3 to 99.1% for individual criteria and from 92.4 to 94.5% for the total score. Kappa coefficients ranged from 0.95 to 0.98 for individual criteria and were 0.97 and 0.96 for the total score, depending on the expert. The ICC for the total score was consistently excellent, reaching 0.99 for both experts.

**Table 3:** Intra-rater reliability for jumping rope assessment for AI model and experts correcting AI-generated scores.

Jumping rope	Percentage of observed agreements (%)	Cohen’s kappa (95% CI)	ICC (95% CI)
Criterion 1			
AI model	100.0	1.00 (1.00–1.00)	
Corrected AI-Expert 1	99.1	0.98 (0.96–1.00)	
Corrected AI-Expert 2	98.7	0.97 (0.94–1.00)	
Criterion 2			
AI model	100.0	1.00 (1.00–1.00)	
Corrected AI-Expert 1	98.7	0.97 (0.94–1.00)	
Corrected AI-Expert 2	98.3	0.96 (0.93–1.00)	
Criterion 3			
AI model	100.0	1.00 (1.00–1.00)	
Corrected AI-Expert 1	98.3	0.96 (0.93–1.00)	
Corrected AI-Expert 2	98.7	0.97 (0.94–1.00)	
Criterion 4			
AI model	100.0	1.00 (1.00–1.00)	
Corrected AI-Expert 1	98.3	0.97 (0.93–1.00)	
Corrected AI-Expert 2	97.9	0.96 (0.92–0.99)	
Criterion 5			
AI model	100.0	1.00 (1.00–1.00)	
Corrected AI-Expert 1	99.1	0.98 (0.95–1.00)	
Corrected AI-Expert 2	97.9	0.95 (0.91–0.99)	
Total			
AI model	100.0	1.00 (1.00–1.00)	1.00 (1.00–0.1.00)
Corrected AI-Expert 1	94.5	0.97 (0.96–0.99)	0.99 (0.99–0.99)
Corrected AI-Expert 2	92.4	0.96 (0.94–0.98)	0.99 (0.98–0.99)

Both experts maintained high consistency between initial and subsequent assessments across all criteria, with only minor, non-systematic discrepancies observed. Differences between sessions ranged from 2 to 5 cases per criterion, and total score consistency was high (expert 1: 223 out of 236 cases; expert 2: 218 out of 236 cases).

## Discussion

This study confirmed the validity and reliability of an AI-enhanced methodology for assessing jumping rope performance within the FUS test framework. The AI model closely aligned with expert evaluations, as shown by high correlation coefficients and near-perfect agreement across both individual criteria and total scores. When refined by expert input, the model’s inter-rater reliability improved further, demonstrating its capacity for iterative enhancement. Intra-rater reliability was consistently high, with the AI model alone achieving perfect agreement (kappa = 1.00, ICC = 1.00); after expert corrections, reliability remained robust, with only a minor reduction. These results support the use of AI systems as accurate, consistent tools for FMS evaluation, with clear implications for research and PE practice.

First, the strong agreement between the AI model and expert scores demonstrates its capacity to replicate expert evaluations with high accuracy and consistency. This advancement addresses long-standing limitations of traditional assessment methods, such as evaluator bias, fatigue-induced errors, and inter-rater variability (Hulteen et al., 2023). The AI system applies standardized criteria uniformly, thereby reducing the influence of subjective judgment and eliminating inconsistencies across evaluators. This is especially valuable in multi-site studies and large-scale educational settings, where consistent conditions are essential (Barnett et al., 2014). Moreover, the model supports post-hoc analysis of video recordings, enhancing transparency and reliability in performance evaluations (Atkinson and Nevill, 1998; Leukel et al., 2023; Ward et al., 2020). These features position the AI model as a viable and scalable alternative to traditional human-led assessments, particularly in contexts where time, training, and resources are constrained.

Second, the AI model shows strong potential for iterative refinement, leveraging performance data to continuously improve its scoring accuracy—an advantage over human experts, who often rely on fixed methods and experiential judgment (Song et al., 2021; Vandevoorde et al., 2022). Unlike human raters, whose perceptual limitations may lead to inconsistency in identifying nuanced movement errors, AI systems can detect subtle deficits with precision. Barnett et al. (2014) illustrated this limitation in their analysis of complex motion sequences like the overhand throw, where evaluators frequently disagreed during rapid movement phases such as the wind-up or follow-through. These discrepancies, compounded by the cognitive load of observing multi-joint actions in real time, underscore the limits of human perception. In contrast, AI systems can process detailed kinematic data objectively and without fatigue, ensuring greater accuracy and consistency in movement evaluation.

Third, the alignment between AI-derived evaluations and expert assessments highlights the system’s adaptability to applied settings, including PE classes. For teachers with limited time or specialized training, the AI model offers a practical and efficient solution, providing consistent evaluations without reliance on subjective judgment. By addressing scoring variability and logistical constraints, it facilitates timely, individualized interventions based on accurate performance feedback. Morley et al. (2019) similarly observed that digital tools enhance assessment efficiency and reduce resource demands, enabling educators to prioritize evidence-based instruction. These benefits position AI-enhanced tools as valuable assets for improving the quality, accessibility, and standardization of motor skill assessments, ultimately supporting physical literacy and motor competence development.

Finally, the validated AI model offers new opportunities to democratize access to high-quality FMS assessments, particularly in resource-limited educational contexts. While this study provides an initial demonstration of the model’s utility, broader implementation could transform PE by enabling scalable, AI-powered evaluation systems. Kok et al. (2020) showed that digital tools, such as self-controlled video feedback, enhance motor learning and self-efficacy by promoting autonomy and self-regulation. Building on this foundation, AI systems may empower students to assess and reflect on their performance independently, reducing reliance on direct teacher instruction while sustaining accuracy and engagement. Moreover, such tools can support peer-to-peer assessments through structured, video-based applications enriched with instructional cues. This collaborative model fosters a motivating, student-centered learning environment and helps accommodate challenges like limited instructional capacity and large class sizes. Through the refinement of traditional assessment practices, AI-enhanced tools have the potential to promote both physical literacy and educational equity. Future research should explore the integration of adaptive feedback features to optimize motor learning and encourage student agency in FMS development.

Inter-rater reliability results confirmed the AI model’s capacity to replicate expert judgments with high consistency, with near-perfect agreement across criteria and excellent ICC values. These outcomes were comparable to or exceeded inter-rater reliability levels reported in studies involving human experts (Makaruk et al., 2023; Zamani et al., 2024), underscoring the system’s potential as a standardized evaluation tool. Notably, previous research has highlighted difficulties even among trained assessors. For instance, Hulteen et al. (2023) reported component-level agreement as low as 40.5%, particularly for tasks like skipping, while Ward et al. (2020) observed criterion-level accuracy ranging from 35 to 100%, often due to attentional lapses and inconsistent interpretation of scoring criteria. Incorporating expert oversight into the AI model improved inter-rater reliability further, suggesting that a hybrid approach—combining algorithmic precision with expert refinement—can elevate assessment quality. This process reduces subjective biases and ensures consistent application of criteria, thereby enhancing both the reliability and scalability of motor skill evaluations in research and applied settings.

Intra-rater reliability analysis reinforced the robustness of the AI model, particularly for longitudinal monitoring of motor skill development. The system achieved perfect agreement across all criteria and total scores, with kappa and ICC values reaching 1.00—a level virtually impossible to attain for human experts in intra-rater FMS assessments. This level of precision supports the use of AI tools for progress tracking, timely intervention, and performance optimization in educational and developmental contexts. Even after expert corrections, reliability remained high, exceeding benchmarks reported in prior studies (Makaruk et al., 2023; Zamani et al., 2024). These findings suggest that AI-assisted assessment can enhance data-driven decision-making in both research and applied practice, while maintaining consistency over time.

Discrepancies between the AI model and expert evaluations were generally minor and varied across criteria, indicating non-systematic biases rather than consistent directional errors. For instance, the AI model occasionally overestimated performance in Criterion 1 by assigning higher scores when participants quickly resumed jumping after an error, overlooking brief interruptions in continuity. In contrast, it adopted a more conservative approach in Criterion 2, underestimating performance due to prolonged ground contact. This bidirectional pattern indicates stochastic variation rather than a systematic scoring bias. Additionally, slight inconsistencies across repeated expert assessments suggest that some variation may stem from the subjective nature of human judgment rather than model limitations. These findings reinforce the importance of ongoing algorithmic refinement to better align with expert standards and ensure robust applicability in real-world assessment contexts.

Several limitations should be acknowledged. First, the model’s accuracy depends on controlled testing conditions, including precise camera alignment, sufficient lighting, and a contrast-enhancing background—which may be challenging to reproduce in certain PE class environments. Second, the study focused solely on jumping rope, which, while informative, limits the generalizability of findings to other FMS with differing biomechanical and coordination demands. Future research should extend the framework to a broader range of skills. Lastly, minor over-and underestimations by the AI in specific criteria highlight the need for continued algorithmic refinement to improve sensitivity to nuanced movement variations and ensure alignment with expert standards.

## Conclusion

This study provides robust evidence supporting the validity and reliability of an AI-enhanced methodology for assessing jumping rope performance within the FUS test framework. The AI model effectively replicated expert-level evaluations, and its performance was further strengthened through expert refinement. These results highlight the system’s potential to overcome key limitations of traditional assessment methods by offering scalable, objective, and consistent evaluations. Beyond its research value, the model presents a practical solution for educational and sport contexts, particularly in settings with limited resources. With further development, such tools may help democratize access to high-quality motor skill assessments and support broader efforts to enhance motor competence and health outcomes across populations.

## Data Availability

The original contributions presented in the study are included in the article/Supplementary material, further inquiries can be directed to the corresponding author.
