# Correction: Artificial intelligence-enhanced assessment of fundamental motor skills: validity and reliability of the FUS test for jumping rope performance

**DOI:** 10.3389/frai.2025.1710897

**Published:** 2025-10-16

**Authors:** Hubert Makaruk, Jared M. Porter, E. Kipling Webster, Beata Makaruk, Paweł Tomaszewski, Marta Nogal, Daniel Gawłowski, Łukasz Sobański, Bartosz Molik, Jerzy Sadowski

**Affiliations:** ^1^Faculty of Physical Education and Health in Biala Podlaska, Józef Piłsudski University of Physical Education in Warsaw, Warsaw, Poland; ^2^Department of Kinesiology, Recreation, and Sport Studies, University of Tennessee, Knoxville, TN, United States; ^3^Faculty of Physical Education, Józef Piłsudski University of Physical Education in Warsaw, Warsaw, Poland; ^4^Artificial Intelligence Department, DG Consulting, Wrocław, Poland; ^5^Department of Research in Artificial Intelligence, Instat sp. z o.o., Wrocław, Poland; ^6^Faculty of Rehabilitation, Józef Piłsudski University of Physical Education in Warsaw, Warsaw, Poland

**Keywords:** motor competence, fundamental movement skills, machine learning, mobile application, physical education

Incorrect text was included in the abstract.

The text has been corrected to read:

**Introduction:** Widespread concerns about children's low fundamental motor skill (FMS) proficiency highlight the need for accurate assessment tools to support structured instruction. This study examined the validity and reliability of an AI-enhanced methodology for assessing jumping rope performance within the Fundamental Motor Skills in Sport (FUS) test.

**Methods:** A total of 236 participants (126 primary school students aged 7–14; 110 university sports students aged 20–21) completed jumping rope tasks recorded via the FUS mobile app integrated with an AI model evaluating five process-oriented performance criteria. Concurrent validity and inter-rater reliability were examined by comparing AI-generated assessments with scores from two expert evaluators. Intra-rater reliability was also assessed through reassessment of video trials after a 3-week interval.

**Results:** Results revealed excellent concurrent validity and inter-rater reliability for the AI model compared with expert ratings (ICC = 0.96; weighted kappa = 0.87). Agreement on individual criteria was similarly high (Cohen's kappa = 0.83–0.87). Expert-adjusted AI scores further improved reliability (ICC = 0.98). Intra-rater reliability was also excellent, with perfect agreement for AI-generated scores (ICC = 1.00; kappa = 1.00).

**Conclusions:** These findings demonstrate that AI-based assessment offers objective, reliable, and scalable evaluation, enhancing accuracy and efficiency of FMS assessment in education and research.

The original version of this article has been updated.

